# Assessment of laparoscopic stomach preserving surgery with sentinel basin dissection versus standard gastrectomy with lymphadenectomy in early gastric cancer–A multicenter randomized phase III clinical trial (SENORITA trial) protocol

**DOI:** 10.1186/s12885-016-2336-8

**Published:** 2016-05-31

**Authors:** Ji Yeon Park, Young-Woo Kim, Keun Won Ryu, Byung-Ho Nam, Young Joon Lee, Sang Ho Jeong, Ji-Ho Park, Hoon Hur, Sang-Uk Han, Jae Seok Min, Ji Yeong An, Woo Jin Hyung, Gyu Seok Cho, Gui Ae Jeong, Oh Jeong, Young Kyu Park, Mi Ran Jung, Hong Man Yoon, Bang Wool Eom

**Affiliations:** Department of Surgery, National Cancer Center, Goyang, Republic of Korea; Department of Cancer Control and Policy, Graduate School of Cancer Science and Policy, National Cancer Center, 323 Ilsan-ro, Ilsandong-gu, Goyang, Gyeonggi-do 10408 Republic of Korea; Center for Gastric Cancer, National Cancer Center, Goyang, Republic of Korea; Department of Surgery, Gyeongsang National University, Jinju, Korea; Department of Surgery, Ajou University School of Medicine, Suwon, Korea; Department of Surgery, Dongnam Institute of Radiological and Medical Science, Busan, Korea; Department of Surgery, Yonsei University School of Medicine, Seoul, Korea; Department of Surgery, Soonchunhyang University College of Medicine, Bucheon, Korea; Department of Surgery, Chonnam National University Hwasun Hospital, Hwasun, Korea; Present address: Department of Surgery, Kyungpook National University Medical Center, Daegu, Korea; Present address: Department of Surgery, Samsung Medical Center, Seoul, Korea

**Keywords:** Phase III clinical trial, Gastric cancer, Sentinel lymph node, Laparoscopic surgery

## Abstract

**Background:**

Along with the marked increase in early gastric cancer (EGC) in the Eastern countries, there has been an effort to adopt the sentinel node concept in EGC to preserve gastric function and reduce the occurrence of postoperative complications. Based on promising results from a previous quality control study, this prospective multicenter randomized controlled phase III clinical trial aims to elucidate the oncologic safety of laparoscopic stomach-preserving surgery with sentinel basin dissection (SBD) compared to a standard laparoscopic gastrectomy.

**Methods/Design:**

This trial is an investigator-initiated, open-label, multicenter randomized controlled phase III trial with a non-inferiority design. Patients diagnosed with a single lesion of clinical stage T1N0M0 gastric adenocarcinoma, with a diameter of 3 cm or less are eligible for the present study. A total of 580 patients (290 per group) will be randomized to either laparoscopic stomach-preserving surgery with SBD or standard surgery. The primary end-point is 3-year disease-free survival (DFS) and the secondary endpoints include postoperative morbidity and mortality, quality of life, 5-year DFS, and overall survival. Qualified investigators who completed the prior quality control study are exclusively allowed to participate in this phase III clinical trial.

**Discussion:**

The proposed trial is expected to verify whether laparoscopic stomach-preserving surgery with SBD achieves similar oncologic outcomes and improved quality of life compared to a standard gastrectomy in EGC patients.

**Trial registration:**

This study was registered at the NIH ClinicalTrial.gov database (NCT01804998) on March 4th, 2013.

## Background

Gastrectomy with extended lymph node dissection has long been considered a standard treatment for gastric cancer to ensure satisfactory long-term survival [1]. As nodal metastasis has a great influence on disease prognosis following curative treatment for gastric cancer, the complete eradication of potential metastatic nodes is essential to reduce loco-regional recurrence and achieve optimal oncologic outcomes.

However, some investigators question whether the standard treatment can be excessive in certain populations with early-stage disease where prophylactic lymph node dissection might play a minor role in terms of curing the disease. The prevalence of lymph node metastasis in early gastric cancer (EGC) is reported to be in the range of 7.7 to 19.4 % [2–4], which means that the remainder of patients are free of nodal metastasis and may unnecessarily undergo an extensive lymphadenectomy at the expense of their quality of life (QOL).

The sentinel node (SN) is defined as the first lymph node to receive lymphatic drainage from the primary tumor, and lymph node metastasis is assumed to initially occur at this site. Many investigators have demonstrated that metastasis via the lymphatic channel occurs in a stepwise manner in malignant melanoma and breast cancer, and SNs can represent the overall metastatic status of the lymph nodes [5, 6]. The accuracy of a sentinel lymph node biopsy is reported to extend over 95 % in breast cancer and melanoma [7–9], and this result provides supporting evidence to obviate unnecessary lymphadenectomy in those with negative SNs, which consequently leads to less frequent postoperative morbidity and improved QOL in patients with breast cancer or malignant melanoma [10–12].

There has been consistent effort over the last decade to apply the SN concept in gastric cancer. As the standard gastrectomy with lymphadenectomy can induce unwanted surgical complications, as well as, long-term nutritional and functional deficits, SN navigation surgery is expected to provide a better QOL in gastric cancer patients by reducing the extent of the surgery with respect to lymph node dissection and gastric resection. However, the clinical application of SN biopsy in gastric cancer has been challenging due to the complicated nature of multidirectional lymphatic drainage in the stomach, and the possibility of skip metastasis [13–15].

Nonetheless, the details of the procedure have gradually evolved, to improve the outcomes of SN detection and evaluation in gastric cancer patients, through trial and error [16]. A recently published multicenter study from Japan demonstrated promising results in terms of the feasibility of SN navigation surgery in gastric cancer patients [17]. However, it is yet to be adopted as routine clinical practice owing to insufficient evidence of oncologic safety compared to conventional surgery.

Therefore, we herein propose a randomized controlled clinical trial (SEntinel Node ORIented Tailored Approach [SENORITA] trial) to elucidate whether stomach-preserving surgery with sentinel basin dissection (SBD) achieves a similar disease-free survival (DFS) rate as the standard gastrectomy, as well as, the impact on postoperative morbidity, mortality, and QOL in patients with EGC.

## Methods/Design

### Study design

**Fig. 1:**
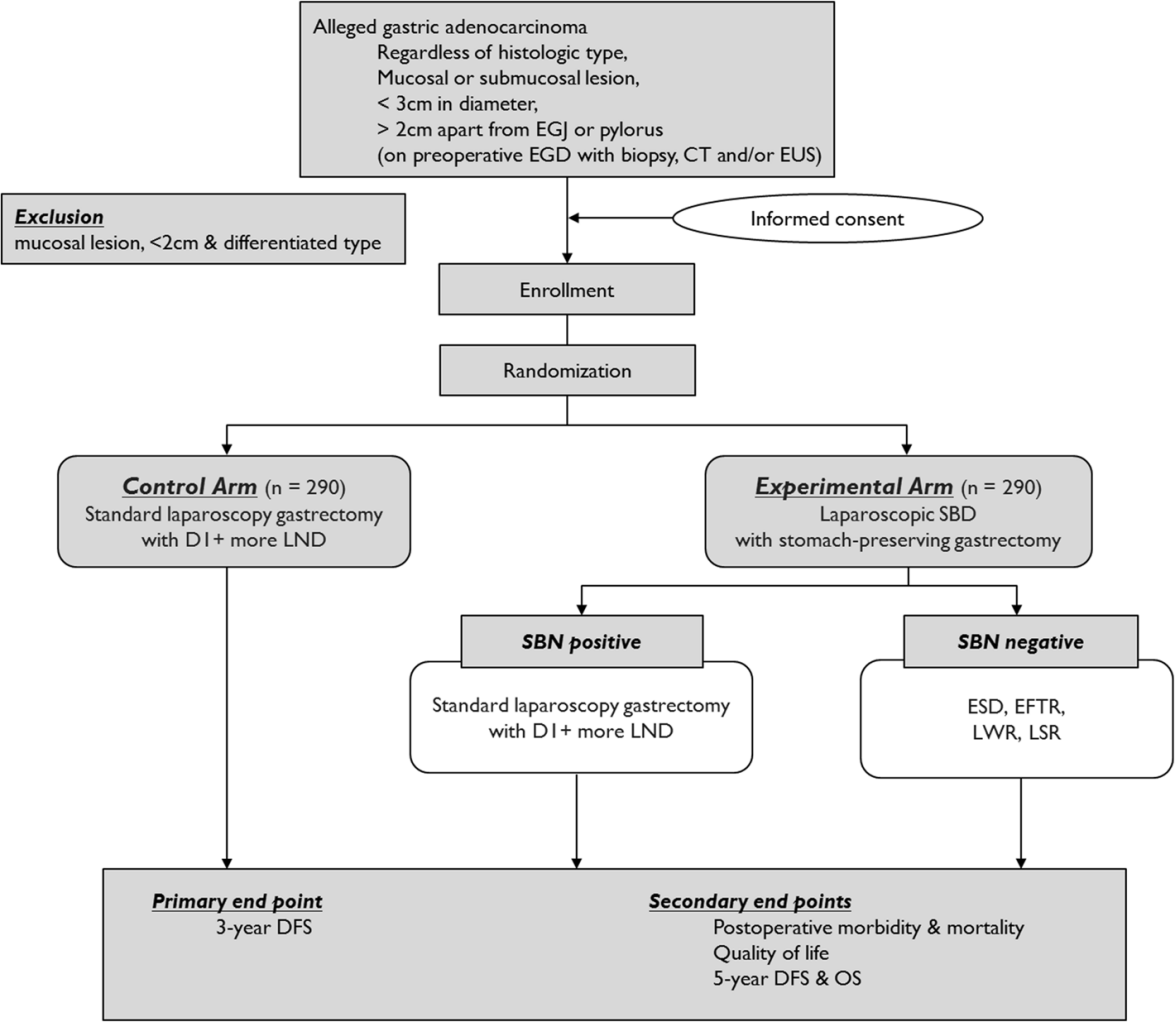
Study scheme of SENORITA trial, inclusion and exclusion criteria, intervention, and end points (EGJ, esophagogastric junction; EGD, esophagogastroduodenoscopy; CT, computed tomography; EUS, endoscopic ultrasonography; LND, lymph node dissection; SBD, sentinel basin dissection; SBN, sentinel basin node; ESD, endoscopic submucosal dissection; EFTR, endoscopic full-thickness resection; LWR, laparoscopic wedge resection; LSR, laparoscopic segmental resection; DFS, disease-free survival; OS, overall survival)

The SENORITA trial is an investigator-initiated, open-labeled, parallel-assigned, multicenter randomized controlled phase III trial. It is schematically described in Fig. 1. This study will involve 7 medical institutions (National Cancer Center, Gyeongsang National University Hospital, Ajou University Hospital, Dongnam Institute of Radiological and Medical Science, Yonsei University Severance Hospital, Soonchunhyang University Bucheon Hospital, and Chonnam National University Hwasun Hospital), which have been qualified to participate in this phase III trial following completion of the prior quality control study (NCT01544413) [18, 19].

The institutional review board (IRB) of the National Cancer Center, Korea has approved this study (IRB No. NCCCTS-13-661). The study has also been approved by the local ethical committee of each participating center (Gyeongsang National University Hospital [2013-06-002], Ajou University Hospital [AJIRB-MED-OBS-13-338], Dongnam Institute of Radiological and Medical Science [D-1304-002-001], Yonsei University Hospital [4-2013-0491], Soonchunhyang University Bucheon Hospital [SCHBC 2013-01-099], and Chonnam National University Hwasun Hospital [CNUHH-2014-051]). Written informed consent will be obtained from all patients prior to patient recruitment. The trial has been registered in the database of clinical trials (NCT01804998). An independent data monitoring committee (IDMC), which is separately organized by independent experts who are not participating in this study, will monitor the clinical trial; the IDMC consists of a surgeon, a gastroenterologist, and a statistician.

### Study population & eligibility criteria

The patient inclusion and exclusion criteria are as follows:

#### The following patients were included in the study

i. Patients with a single lesion of histologically confirmed adenocarcinoma in the stomach in preoperative endoscopic biopsy
ii. Patients with clinical stage of T1N0M0 gastric cancer according to the American Joint Committee for Cancer (AJCC) 7^th^ edition [20] (determined by preoperative endoscopy and computed tomography and/or endoscopic ultrasound)
iii. Patients with a gastric cancer of less than 3 cm as the longest diameter
iv. Patients with a gastric cancer at least 2 cm apart from the pylorus or the cardia
v. Patients who plan to undergo laparoscopic surgery
vi. Patients aged > 20 and < 80 years
vii. Patients with an Eastern Cooperative Oncology Group (ECOG) performance status of 0 or 1
viii. Patients who agree to participate in the clinical study through informed consent

#### The following individuals were excluded from the study

i. Patients with a lesion satisfying the absolute indications of endoscopic resection (<2 cm, mucosal lesion, differentiated type)
ii. Patients who are not indicated for surgical treatment due to serious cardiovascular or pulmonary disease
iii. Pregnant women
iv. Patients with a past history of drug-related anaphylactic reaction, prior upper abdominal surgery (except for laparoscopic cholecystectomy) or radiation therapy
v. Patients diagnosed with other malignancy within 5 years

### Randomization & allocation

The web-based clinical trial management system (eVelos System; Velos, Inc., Fremont, CA: http://eresearch.ncc.re.kr/velos/jsp/ereslogin.jsp) at the Clinical Research Coordination Center (CRCC) within the National Cancer Center, Korea, will coordinate the study and handle the data analysis. As soon as written informed consent is obtained from the eligible patient, the patient will be registered in the system and randomly allocated to one of the two surgical groups: laparoscopic SBD with stomach preserving surgery or standard laparoscopic gastrectomy with D1+ or more lymphadenectomy according to the Japanese gastric cancer treatment guidelines [1]. To reduce potential bias and confounding, participants will be further stratified based on the institution where the procedure is performed, depth of tumor invasion (mucosa vs. submucosa), and size (≤2 cm vs. > 2 cm) of the primary tumor. The random block size permutation method was used to generate the initial randomization sequence, and the randomization task will be centrally coordinated by the CRCC.

All registered patients will undergo a routine preoperative evaluation, including full laboratory tests, electrocardiogram, chest X-ray, pulmonary function test, and assessment for tumor markers.

### Intervention

#### Experimental group

##### Procedures for SBD

A detailed description of the SBD procedure is provided in our preceding report on the quality control study for the SENORITA trial [19]. Briefly, a mixture of indocyanine green (IGC; Diagnogreen®, Daiichi-Sankyo Co., Ltd., Japan; 2 mL, 5 mg) and radiolabeled human serum albumin (Tc99m-HSA; 2 mL, 0.1 mCi/mL) is used as a tracer to detect the SNs. The tracer is uniformly prepared at the reference center and delivered to the participating institutions. A 4-mL volume of the dual tracer is injected into the submucosal layer in 4 quadrants of the primary tumor via an intraoperative endoscopic approach. After 15 min of the endoscopic tracer injection, the sentinel basins containing SNs (green or hot) are carefully dissected and retrieved from the surgical field.

The harvested sentinel basins are then dissected to isolate lymph nodes, whilst in the operating room. All the isolated lymph nodes from the sentinel basins, defined as sentinel basin nodes (SBN), are classified into hot nodes (HN: radioactive nodes), green nodes (GN: stained nodes), both hot and green nodes (HGN), and basin nodes (BN: nodes within the sentinel basins, but neither hot nor green), labeled with the respective lymph node station numbers, and sent to the pathologist for intraoperative frozen section evaluation.

##### Intraoperative & postoperative pathologic evaluation

The harvested nodes from the sentinel basins are histologically examined intraoperatively with hematoxylin and eosin (H&E) staining using 1 representative cut plane of a frozen section for lymph nodes less than 4 mm. For nodes thicker than 4 mm, a slice will be made at a 2-mm interval parallel to the long axis so as not to miss macrometastasis. If all the harvested SBNs are tumor-free, stomach-preserving primary tumor resection is then carried out in accordance with the suggested procedure in the previous publication [4]. The detailed methods of primary tumor control are shown in Table 1. The resection margins of the specimen containing the primary tumor will be intraoperatively evaluated with frozen section examination as well; and patients with margin involvement will undergo further resection to achieve negative margins, or be converted to the standard surgery depending on the intraoperatively measured tumor size and margin status. However, specimens from the endoscopic resection will be reserved for permanent pathology.

**Table 1:** Recommended methods of primary tumor control in the SENORITA trial

	Mucosa		Submucosa	
	≤20 mm	21–30 mm	≤20 mm	21–30 mm
Differentiated type	Exclusion	ESD or LWR/EFTR or LSR	LWR/EFTR or LSR	LSR
Undifferentiated type	ESD or LWR/EFTR or LSR	LSR	LWR/EFTR or LSR	LSR

*ESD* endoscopic submucosal dissection, *LWR* laparoscopic wedge resection, *EFTR* endoscopic full-thickness resection, *LSR* laparoscopic segmental resection

After the surgery, those SBNs proven tumor-free in the intraoperative frozen section examination will be re-evaluated. For permanent histologic evaluation, 1 section of the paraffin-embedded SBNs is stained with H&E and cytokeratin immunohistochemistry (IHC). The nodes are further examined with H&E stains for 3 deeper step sections at 200-μm intervals. When the intraoperative report is proven to be a false negative, or the SBNs are found by way of enhanced procedures to have intraoperatively unnoticed macrometastasis in the paraffin-embedded sections, the patient will be designated to undergo reoperation with the standard surgery. Conversely, watchful observation will be prescribed for patients with micrometastasis or isolated tumor cells (ITC) only in paraffin-embedded sections.

Meanwhile, in cases where macro- or micrometastasis is detected in the frozen sections during intraoperative pathologic examination, the surgery will be converted immediately to a standard gastrectomy with lymphadenectomy, as suggested in the Japanese gastric cancer treatment guidelines.[1]

#### Control group

The patients allocated to the control group will undergo standard laparoscopic gastrectomy, which includes laparoscopic distal gastrectomy, laparoscopic total gastrectomy, laparoscopic proximal gastrectomy, and laparoscopic pylorus-preserving gastrectomy, with D1+ or more lymphadenectomy [1].

### Postoperative follow-up schedule

All patients enrolled in this trial will be followed up regularly at stated intervals after the surgery. During the follow-up visits, patients will undergo laboratory tests, endoscopy, abdominal computed tomography, and QOL evaluation as described in Table 2.

**Table 2:** Summary of the follow-up visit schedule and assessed parameters at each time point

	Preoperative	1 M	3 M	6 M	12 M	18 M	24Y	30 M	36 M	42 M	48 M	54 M	60 M
EGD	*			*	*	*	*	*	*		*		*
CT	*			*	*	*	*	*	*		*		*
Laboratory	*	*	*	*	*	*	*	*	*	*	*	*	*
QOL	*		*		*		*		*				

*EGD* esophagogastroduodenoscopy, *CT* computed tomography, *QOL* quality of life

* Evaluation at the desginated time point

### Outcome measurement

#### Primary outcome

The primary endpoint of the SENORITA trial is 3-year DFS of laparoscopic stomach-preserving surgery with SBD compared to that of conventional laparoscopic gastrectomy with lymphadenectomy in gastric cancer patients diagnosed as clinical stage T1N0M0. To assess DFS in this trial, the criteria for the event is as follows: recurrence of the primary tumor at resection margins, metachronous cancer development at the remnant stomach, histologically proven or radiologically apparent recurrence in the peritoneal cavity including intraabdominal lymph nodes, distant metastasis, newly developed malignancy in other organs, and other cause of death.

#### Secondary outcomes

The secondary endpoints of this trial include postoperative morbidity and mortality, QOL, and 5-year DFS and overall survival. Postoperative morbidity and mortality occurring within 30 days after the surgery will be reported and graded according to the modified Clavien-Dindo severity classification [21].

QOL will be assessed by the validated Korean version of the European Organization for Research and Treatment of Cancer (EORTC) questionnaires. The enrolled patients will be requested to fill out the core questionnaire (QLQ-C30) and the gastric cancer-specific module (QLQ-STO22) annually up to 3 years after the surgery [22, 23].

The trial will be assessed primarily based on the 3-year DFS. In the likelihood that the number of target events is insufficient for statistical analysis after the 3 years of follow-up, the 5-year DFS and overall survival will be reported as an alternative at the end of the study.

### Sample size calculation

The primary endpoint of this clinical trial is 3-year DFS of patients with gastric cancer of clinical stage T1N0M0. The required sample size was calculated based on a non-inferiority design assuming 97 % of patients achieve 3-year DFS following standard surgery. The margin of non-inferiority was 5 % and type 1 error was set at 0.05 with 80 % statistical power. As such, a sample size of 261 in each group with 24 target events is required. After considering a potential dropout rate of 10 % over the follow-up period, the final sample size is estimated as 290 patients in each study group (580 patients in total).

### Data management

All patient data collected during this clinical trial will be maintained as an electronic case report form (eCRF) in a web-based central platform (eVelos System: Velos, Inc., Fremont, CA) at the CRCC. The management team at the CRCC will review the eCRFs, and queries will be sent out to each investigative site regularly. Data monitoring will also be conducted by way of site visits. The data will be managed and analyzed according to the study protocol.

### Safety assessment

When a total of 50 patients are enrolled and the allocated surgeries are completed, safety of the experimental intervention or laparoscopic stomach-preserving surgery with SBD, will be evaluated in terms of the occurrence of postoperative complications. Subsequently, the IDMC will evaluate the results of the safety analysis and provide a recommendation as to whether the trial should proceed.

Any serious adverse events (SAEs) will be documented in the medical records, as well as, in the eCRF and reported to the IRB by the responsible investigator, in accordance with the local regulations. SAE is defined as a postoperative complication of Grade III or above based on the Clavien-Dindo classification system [21], or readmission within one month after the surgery.

### Interim analysis

When the number of events reaches 12 (50 % of the expected number of events), an interim analysis will be performed to identify any evidence of definite inferiority of the experimental intervention. The IDMC will subsequently evaluate whether the trial should be continued or terminated based on the results of the interim analysis.

### Statistical analysis

Non-inferiority of the experimental study arm will be claimed if the lower confidence limit of 3-year DFS in the experimental study group exceeds 92 %. For survival analyses, the Cox proportional hazards model will be used. Kaplan-Meier curves will be used for survival curve estimation. Categorical variables will be analyzed by the Pearson’s *χ*^2^ test or Fisher’s exact test and continuous variables will be evaluated by the Student’s t-test or appropriate non-parametric method as required. The level of statistical significance will be set at 5 %.

## Discussion

In recent years there has been a rapid increase in the detection of early stage gastric cancer in Korea and Japan, and the number of long-term survivors has markedly increased accordingly [24]. In an effort to preserve the patient’s post-treatment QOL, minimally invasive procedures, such as laparoscopic surgery, have gained widespread popularity in gastric cancer treatment [25]. Endoscopic resection, which is regarded as less invasive for preserving physiological gastric function, is one such alternative option for carefully selected patients with EGC and very low risk of lymph node metastasis. However, the range of its application is limited, and an extension of the eligible criteria is still debated because of the possibility of neglecting a metastatic lymph node.

Accurate prediction of lymph node metastasis is mandatory in order to reduce the extent of surgery, without hampering oncologic safety in EGC patients. Presently, however, no modality is capable of making a definite diagnosis of nodal metastasis before surgical resection. Although the technology used for preoperative evaluation, including endoscopy, abdominal CT, and endoscopic ultrasonography has markedly developed so far, it still has limited accuracy for nodal staging in gastric cancer patients. In recent decades, significant effort has also been undertaken to improve biomedical imaging technology for noninvasive detection of microscopic metastases in lymph nodes; this involves multiple imaging modalities including ultrasonography, magnetic resonance images, and positron emission tomography, as well as, novel technologies such as nanotechnology and photoacoustic imaging [26–28]. However, these promising techniques still require further investigation to have an impact in clinical practice. Alternatively, the SN concept, despite some level of invasiveness, is expected to facilitate the avoidance of extensive lymph node dissection, and subsequently to preserve physiologic function in relevant patients with metastatic node-free gastric cancer, as long as it is proven to be feasible and safe.

Many investigators have evaluated the applicability of SN navigation surgery in gastric cancer. The results are inconsistent across studies in terms of the accuracy and sensitivity of SN biopsy for detecting nodal metastasis in gastric cancer; however, most of these studies were conducted with a small population at a single center [29]. Nonetheless, a series of recent studies have reported the feasibility of SN navigation surgery in EGC patients [17, 30–35]. Therefore, we herein propose a multicenter randomized clinical trial primarily to elucidate the oncologic safety of SBD with stomach-preserving surgery compared to the standard laparoscopic gastrectomy with lymphadenectomy.

The most challenging aspect of designing a randomized controlled trial involving surgical procedures is that it is difficult to blind the surgeons and patients as to the intervention. Moreover, the ethical concerns with regards to the blinding of patients must also be considered. Therefore, the primary end-point should be a purely objective variable, such as DFS, as in this clinical trial, to minimize potential bias caused by non-blinding. Secondly, the surgical procedure per se is inevitably operator-dependent and can vary among the participating surgeons. As such, it would be challenging to extrapolate the results of a single-institution trial to other centers. A multicenter trial is also associated with biases, such as differences in operative skill and experience, as well as, in perioperative care among participants. The tendency toward such bias would certainly become stronger when the procedure is more complicated and involves investigators from different departments. Therefore, we obtained in-depth advice from experts in the field before the development of this study protocol. We also conducted a quality control study prior to the initiation of this phase III trial to qualify participating institutions [19]. A detailed step-by-step checklist was provided to investigators participating in the previous quality control study. It was recommended that the checklist be completed for at least 10 patients per surgeon to overcome the learning experience. Repetitive discussion during this quality control study allowed participating investigators to achieve consensus and standardize the specific procedures outlined in this subsequent phase III SENORITA trial protocol.

In conclusion, amid the consistent effort to adopt the SN concept in gastric cancer, the proposed SENORITA trial represents a multicenter randomized controlled trial to elucidate the oncologic safety, as well as, postoperative QOL following laparoscopic SBD with stomach-preserving surgery compared to the standard laparoscopic gastrectomy with lymph node dissection in EGC patients. We believe that this trial would significantly contribute to the evolution of surgical practice in EGC in the future.

## Trial status

A total of enrollment period is presumed to be 4 years and the patients will be followed up for 5 years. The trial is open for recruitment since March 2013 and currently recruiting.

## Abbreviations

AJCC: American Joint Committee for Cancer
BS: basin node
CRCC: clinical research coordination center
DFS: disease-free survival
ECOG: Eastern Cooperative Oncology Group
eCRF: electronic case report form
EGC: early gastric cancer
EORTC: European Organization of Research and Treatment of Cancer
GN: green node
H & E: hematoxylin and eosin
HGN: hot and green node
HN: hot node
ICG: indocyanine green
IDMC: independent data monitoring committee
IHC: immunohistochemistry
IRB: institutional review board
ITC: isolated tumor cell
QOL: quality of life
SAE: serious adverse event
SBD: sentinel basin dissection
SBN: sentinel basin node
SENORITA: sentinel node oriented tailored approach
SN: sentinel node
